# YpeB dimerization may be required to stabilize SleB for effective germination of *Bacillus anthracis* spores

**DOI:** 10.1186/s12866-019-1544-1

**Published:** 2019-07-26

**Authors:** Cameron V. Sayer, David L. Popham

**Affiliations:** Department of Biological Sciences, Virginia Tech, 926 West Campus Drive, Blacksburg, VA 24061 USA

**Keywords:** *Bacillus*, Endospore, Spore, Germination, Cortex

## Abstract

**Background:**

*Bacillus* cells faced with unfavorable environmental conditions undergo an asymmetric division process ultimately leading to the formation of the bacterial spore. In some instances the spore serves as an infectious agent; such is the case with the spore of *Bacillus anthracis* and the disease anthrax. Spores are resistant to a variety of environment conditions including traditional decontamination techniques due to the formation of specialized cellular structures. One such structure, the spore cortex, is a thick layer of modified peptidoglycan that contributes to spore dormancy through maintenance of the dehydrated state of the spore core. During spore germination, degradation of the cortex is required to facilitate complete hydration of the core and a return to vegetative growth. Degradation of the cortex is accomplished through the action of germination-specific lytic enzymes. One of these enzymes, SleB, has been previously shown to require the presence of the YpeB protein for its stable incorporation and subsequent function in spores of *B. anthracis.* The focus of the present study is to identify protein interactions of YpeB through in vivo chemical cross-linking and two-hybrid analysis.

**Results:**

Conserved residues within YpeB PepSY domains were altered to facilitate implementation of a site-specific chemical cross-linker, 4-Azidophenacyl bromide. Analyses of crosslinked-spore extracts suggests that YpeB exists as a dimer or larger multimer within the spore, potentially mediated through interactions of the C-terminal domains. Spores expressing stable truncated forms of YpeB were crosslinked and corresponding truncated dimers were detected. Further characterization of individual YpeB domains using bacterial two-hybrid analysis indicated a possible role for both N-and C-terminal domains in YpeB oligomerization.

**Conclusions:**

The YpeB protein likely exists as dimer or higher-order multimer in the dormant spore. Both the N- and C-terminal YpeB domains contribute to multimerization. SleB likely also exists as an oligomer, and SleB and YpeB may be found together within a protein complex. Disassembly of this complex during spore germination likely allows SleB to become active in spore cortex degradation. Further study of this protein complex may contribute to the development of methods to inhibit or stimulate germination, allowing more effective spore decontamination.

**Electronic supplementary material:**

The online version of this article (10.1186/s12866-019-1544-1) contains supplementary material, which is available to authorized users.

## Background

*Bacillus anthracis* has the potential to cause widespread illness and severe disease through multiple routes of infection. As with many other disease-causing endospore-producing bacterial species, the bacterial endospore serves as the infectious agent of the disease anthrax [1]. This is especially problematic because the inherent resistance characteristics of bacterial spores render many standard decontamination methods ineffective [1–3]. The greatest factor in maintenance of spore resistance properties is preservation of the metabolically dormant and dehydrated state of the spore core [4]. Dormancy is maintained by specialized spore structures including the inner spore membrane and cortex peptidoglycan, and high spore core concentrations of Ca^2+^-dipicolinic acid (DPA). [2, 3, 5]. These factors contribute to the overall threat that *B. anthracis* poses, especially as a bioterrorism agent.

When the dormant spore senses an environment with favorable nutrient availability, such as within a host, it will rapidly germinate, returning to a vegetative growth state. Germination is initiated following sensing of germinants by receptors at the inner spore membrane, after which large stores of Ca^2+^-DPA are released from the spore core and partial rehydration of the core begins [2]. The spore cortex is then depolymerized, facilitating complete hydration of the core and a return to a vegetative growth state [2]. Completion of germination of *B. anthracis* within the host is required for production of the anthrax toxins and ultimately progression of the disease [1].

The cortex is degraded by germination specific lytic enzymes (GSLEs). *B. anthracis* encodes four of these enzymes, but the majority of the cortex degradation has been demonstrated to be completed through the action of partially redundant enzymes SleB and CwlJ1 [6, 7]. These enzymes specifically recognize modified muramic-δ-lactam [8–13], which is found uniquely in spore cortex peptidoglycan [14, 15]. CwlJ1 is localized to the spore coat layer and has been shown to be activated by the release of Ca^2+^-DPA [2, 16, 17].

In *Bacillus* species, both SleB and YpeB are expressed from a conserved operon and this is also true of several *Clostridium* strains [18]. Previous studies have determined that SleB and YpeB co-localize to the inner spore membrane as well as potentially to a second location near the outside of the cortex [17, 19]. Further studies have determined that SleB and YpeB are co-dependent, requiring one another for stable incorporation within the dormant spore in both *B. subtilis* and *B. anthracis* [17, 20–22]. SleB and YpeB are expressed under the control of σ^G^ and are translocated across the inner spore membrane via N-terminal signal sequences [9, 19, 22, 23]. The signal sequence of YpeB is not predicted to be cleaved, leaving YpeB anchored to the inner spore membrane, while SleB is expressed in its mature form, with signal sequence removed, within the dormant spore [19, 23–25]. Given the co-localization, co-dependency, and that SleB is present but held inactive in the dormant spore, it has been theorized that YpeB and SleB interact in some manner to stabilize one another within the dormant spore [20]. Previous studies have implicated a role for both N-terminal (residues 21–202) and C-terminal (residues 203–446) regions of YpeB in interactions with SleB [20, 21]. It has been demonstrated that the N-terminal domain of YpeB was most effective in inhibiting SleB activity in vitro [21], while a region of YpeB beyond the first PepSY domain is required for SleB incorporation within the dormant spore [20].

The goal of the current study was to further characterize the relationship between YpeB and SleB within the dormant spore. In vivo peptide cross-linking was used to study potential interactions of YpeB, identifying interactions that may form only within the unique environment of the dormant spore. Bacterial two-hybrid analysis was used to detect domain-specific interactions. Both methods indicate YpeB oligomer formation, which may be required for stable incorporation of SleB and subsequent germination of the *Bacillus* spore.

## Results

### In vivo site-directed cross-linking of YpeB in *B. anthracis* dormant spores

Previous work has highlighted the importance of the YpeB C-terminal domain (203–446), specifically residues beyond the first PepSY domain, for stabilization of SleB in the developing spore [20]. Interactions of the YpeB C-terminal domains within the dormant spore were further characterized by employing in vivo amino acid-specific chemical cross-linking. Guided by homology modeling of the YpeB C-terminal domain to that of the metalloprotease Vibriolysin [26], several amino acid residues were selected as potential interaction sites. Residues were chosen based on the following criteria: predicted to be surface exposed, critical in PepSY domain interactions in Vibriolysin, and/or conserved in *ypeB* orthologs. Each of the selected residues was then mutagenized, substituting the wildtype codon with that for cysteine, and YpeB-cysteine alleles were recombined into the chromosome of a *∆ypeB* strain. These alleles also carried a C-terminal hexa-histidine tag for protein purification purposes. This tag has previously been shown to not interfere with YpeB function [20]. The functionality of the YpeB-Cys proteins were verified by examination of the abundance of YpeB and SleB in the dormant spores, quantification of optical density (OD) loss during spore germination, and observation of YpeB proteolysis to stable C-terminal products during germination. All YpeB-Cys alleles were very similar to the wild type (WT) in all of these assays. Specifically, the two alleles utilized for further studies, *ypeBS358C-6His* and *ypeBK437C-6His* were nearly identical to the wild type in these regards (Additional file 1). Interestingly, these two YpeB-Cys alleles allowed normal OD loss during germination but resulted in slow spore outgrowth, potentially indicating an additional YpeB effect on germination.

**Fig. 1:**
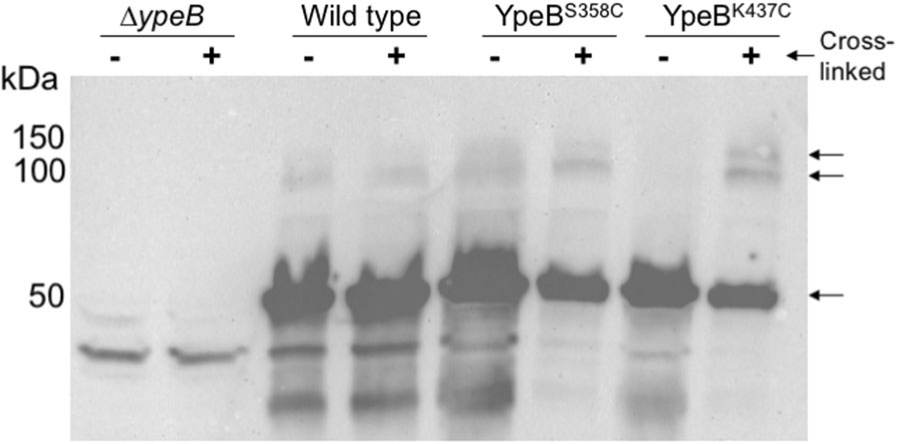
YpeB-Cys can be cross-linked in spores. Ten optical density units of decoated dormant spores were incubated with 5 mM APB for 30 min at 37 °C in reduced light and then irradiated with UV light for 15 min. Cross-linked spores were lyophilized and mechanically broken, and proteins were extracted. Whole spore lysates were then visualized via Western blot using anti-YpeB antibodies [20]. The positions of protein size markers are indicated on the left. The YpeB monomer is indicated by the arrow at 50 kDa, higher migrating bands are indicated by arrows at ~ 100 and 150 kDa in YpeB-Cys cross-linked samples

Sulfhydryl specific cross-linking was conducted using the cross-linker p-azidophencyl bromide (APB), which is a heterobifunctional cross-linker with a sulfhydryl specific α-bromo-ketone motif in addition to a non-specific photoactivatable azide, separated by a spacer arm of 9 Å. In total, 12 YpeB-Cys allele-carrying strains, corresponding to 12 different amino acid substitutions across the C-terminal PepSY domains (Table 1), were created and tested in site-specific cross-linking schemes of dormant spores. Of the 12 alleles tested, those encoding Cys substitutions for residues 358 and 437 revealed higher migrating complexes of roughly ~ 100 kDa in anti-YpeB western blots of dormant whole spore extracts following APB cross-linking (Fig. 1). It was theorized that because these higher migrating bands appeared at roughly double the mass of the YpeB monomer (~ 50 kDa) that these complexes might contain a cross-linked YpeB dimer. Only a small percentage of the YpeB molecules became cross-linked, presumably due to a low efficiency of cross-linker modification of the YpeB-Cys residues (amid competition by native Cys residues in the spore coats) and potentially low efficiency of the non-specific cross-linking by the photoactivatable azide. Western blots using anti-SleB antibodies revealed no bands representing potential cross-linked YpeB-SleB complexes.

**Table 1:** Bacterial strains and plasmids

Strain	Genotype	Construction^a^	Source
*B. anthracis*			
Sterne 34F2	pXO1^+^ pXO2^−^		P. Hanna
DPBa89	∆*ypeB*	pDPV392 > 34F2	[20]
DPBa127	∆*ypeB*::pDPV424 (YpeB_1–446_-His_6_ Er^R^)	pDPV424 > DPBa89	[20]
DPBa204	∆*ypeB*::pDPV476 (YpeB^K437C^-His_6_ Er^R^)	pDPV476 > DPBa89	This study
DPBa205	∆*ypeB*::pDPV477 (YpeB^S358C^-His_6_ Er^R^)	pDPV477 > DPBa89	This study
DPBa210	∆*ypeB*::pDPV478 (YpeB^T328C^-His_6_ Er^R^)	pDPV478 > DPBa89	This study
DPBa211	∆*ypeB*::pDPV479 (YpeB^M282C-^His_6_ Er^R^)	pDPV479 > DPBa89	This study
DPBa212	∆*ypeB*::pDPV480 (YpeB^Y339C^-His_6_ Er^R^)	pDPV480 > DPBa89	This study
DPBa213	∆*ypeB*::pDPV481 (YpeB^E314C^-His_6_ Er^R^)	pDPV481 > DPBa89	This study
DPBa214	∆*ypeB*::pDPV482 (YpeB^V355C^-His_6_ Er^R^)	pDPV482 > DPBa89	This study
DPBa215	∆*ypeB*::pDPV483 (YpeB^V324C^-His_6_ Er^R^)	pDPV483 > DPBa89	This study
DPBa216	∆*ypeB*::pDPV484 (YpeB^T420C^-His_6_ Er^R^)	pDPV484 > DPBa89	This study
DPBa217	∆*ypeB*::pDPV485 (YpeB^A342C^-His_6_ Er^R^)	pDPV485 > DPBa89	This study
DPBa218	∆*ypeB*::pDPV486 (YpeB^V435C^-His_6_ Er^R^)	pDPV486 > DPBa89	This study
DPBa219	∆*ypeB*::pDPV487 (YpeB^Q438C^-His_6_ Er^R^)	pDPV487 > DPBa89	This study
DPBa220	∆*ypeB*::pDPV488 (YpeB_∆25–203_ K437C-His_6_ Er^R^)	pDPV488 > DPBa89	This study
DPBa221	pDPV488 (YpeB_∆25–203_ K437C-His_6_ Er^R^)	pDPV488 > 34F2	This study
DPBa222	∆*ypeB*::pDPV489 (YpeB_∆25–203_ S358C-His_6_ Er^R^)	pDPV489 > DPBa89	This study
DPBa223	pDPV489 (YpeB_∆25–203_ S358C-His_6_ Er^R^)	pDPV489 > 34F2	This study
*E.coli*			
BTH101			Euromedex
DPVE545	pKT25-zip + pUT18C-zip	pKT25-zip + pUT18C-zip > BTH101	Euromedex
DPVE546	pKT25 + pUT18C		This study
DPVE547	p25-YpeB_21–446_ + p18-YpeB_21–446_	pDPV494 + pDPV490 > BTH101	This study
DPVE548	p25-YpeB_21–446_ + p18-YpeB_21–202_	pDPV494 + pDPV491 > BTH101	This study
DPVE549	p25-YpeB_21–446_ + p18-YpeB_203–446_	pDPV494 + pDPV492 > BTH101	This study
DPVE550	p25-YpeB_21–446_ + p18-SleB_125–253_	pDPV494 + pDPV493 > BTH101	This study
DPVE551	p25-YpeB_21–202_ + p18-YpeB_21–446_	pDPV495 + pDPV490 > BTH101	This study
DPVE552	p25-YpeB_21–202_ + p18-YpeB_21–202_	pDPV495 + pDPV491 > BTH101	This study
DPVE553	p25-YpeB_21–202_ + p18-YpeB_203–446_	pDPV495 + pDPV492 > BTH101	This study
DPVE554	p25-YpeB_21–202_ + p18-SleB_125–253_	pDPV495 + pDPV493 > BTH101	This study
DPVE555	p25-YpeB_203–446_ + p18-YpeB_21–446_	pDPV496 + pDPV490 > BTH101	This study
DPVE556	p25-YpeB_203–446_ + p18-YpeB_21–202_	pDPV496 + pDPV491 > BTH101	This study
DPVE557	p25-YpeB_203–446_ + p18-YpeB_203–446_	pDPV496 + pDPV492 > BTH101	This study
DPVE558	p25-YpeB_203–446_ + p18-SleB_125–253_	pDPV496 + pDPV493 > BTH101	This study
DPVE559	p25-SleB_125–253_ + p18-YpeB_21–446_	pDPV497 + pDPV490 > BTH101	This study
DPVE560	p25-SleB_125–253_ + p18-YpeB_21–202_	pDPV497 + pDPV491 > BTH101	This study
DPVE561	p25-SleB_125–253_ + p18-YpeB_203–446_	pDPV497 + pDPV492 > BTH101	This study
DPVE562	p25-SleB_125–253_ + p18-SleB_125–253_	pDPV497 + pDPV493 > BTH101	This study
Plasmids			
pBKJ236			[27]
pKT25-zip			Euromedex
pUT18C-zip			Euromedex
pKT25			Euromedex
pUT18C			Euromedex
pDPV392		pBKJ236::∆*ypeB*	[20]
pDPV424		pBKJ236::∆*sleB ypeB*_1–446_-His_6_	[20]
pDPV448		pBKJ236::∆*sleB ypeB*_∆*25–203*_-His_6_	[20]
pDPV476		pBKJ236::∆*sleB ypeB*^K437C^-His_6_	This study
pDPV477		pBKJ236::∆*sleB ypeB*^S358C^-His_6_	This study
pDPV478		pBKJ236::∆*sleB ypeB*^T328C^-His_6_	This study
pDPV479		pBKJ236::∆*sleB ypeB*^M282C^-His_6_	This study
pDPV480		pBKJ236::∆*sleB ypeB*^Y339C^-His_6_	This study
pDPV481		pBKJ236::∆*sleB ypeB*^E314C^-His_6_	This study
pDPV482		pBKJ236::∆*sleB ypeB*^V355C^-His_6_	This study
pDPV483		pBKJ236::∆*sleB ypeB*^V324C^-His_6_	This study
pDPV484		pBKJ236::∆*sleB ypeB*^T420C^-His_6_	This study
pDPV485		pBKJ236::∆*sleB ypeB*^A324C^-His_6_	This study
pDPV486		pBKJ236::∆*sleB ypeB*^V435C^-His_6_	This study
pDPV487		pBKJ236::∆*sleB ypeB*^Q438C^-His_6_	This study
pDPV488		pBKJ236::∆*sleB ypeB*_∆25–203_ K437C-His_6_	This study
pDPV489		pBKJ236::∆*sleB ypeB*_∆25–203_ S358C-His_6_	This study
pDPV490		pUT18C::*ypeB*_21–446_	This study
pDPV491		pUT18C::*ypeB*_21–202_	This study
pDPV492		pUT18C::*ypeB*_203–446_	This study
pDPV493		pUT18C::*sleB*_125–253_	This study
pDPV494		pKT25::*ypeB*_21–446_	This study
pDPV495		pKT25::*ypeB*_21–202_	This study
pDPV496		pKT25::*ypeB*_203–446_	This study
pDPV497		pKT25::*sleB*_125–253_	This study

^a^ > indicates transformation of the indicated plasmid into the indicated strain

### In vivo site directed cross-linking of YpeB_∆25–203_ dormant spores

**Fig. 2:**
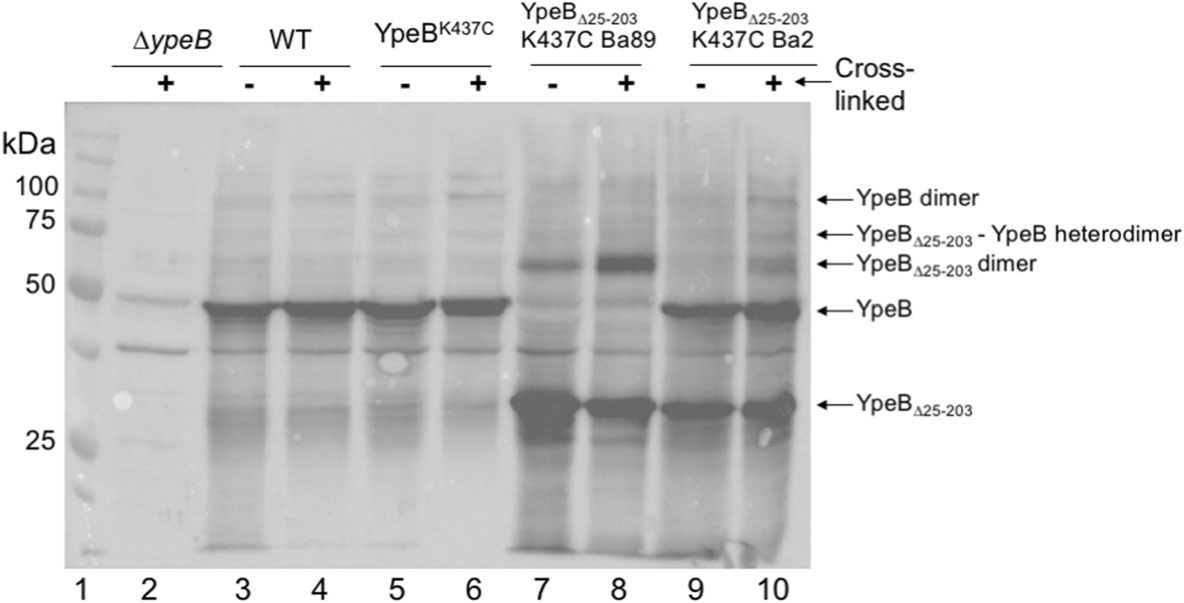
YpeB_∆25–203_-Cys can be cross-linked in spores. Ten optical density units of decoated dormant spores were cross-linked with APB as described previously. Cross-linked spores were lyophilized and mechanically broken, and proteins were extracted. Whole spore lysates were then visualized via western blot using anti-YpeB antibodies [20]. The positions of protein size markers (lane 1) are indicated on the left. YpeB monomer is indicated by the arrow at 50 kDa. YpeB_∆25–203_ monomer is indicated at roughly ~ 30 kDa. YpeB multimers are visualized in YpeB^K437C^ (lane 6) migrating at 100 and 150 kDa. YpeB_∆25–203_ multimers are indicated in YpeB_∆25-203_K437C (lane 8) migrating ~ 60 kDa. A putative YpeB-YpeB_∆25–203_ heterodimer (lane 10) is indicated at ~ 80 kDa, in addition to homodimers identified in previous lanes

**Fig. 3:**
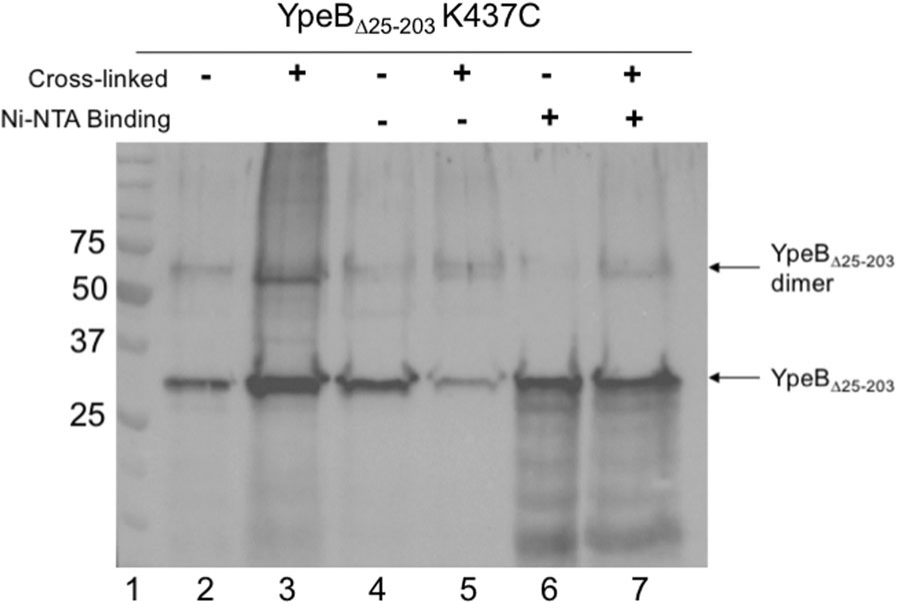
Column-bound YpeB_∆25–203_ K437C cross-linked complex. Two hundred optical density units of decoated dormant spores were cross-linked with APB. Cross-linked spores were lyophilized and mechanically broken. Proteins were extracted with 8 M urea binding buffer for 2 h. Spore lysates (lanes 2–3) were then passed over a Ni^2+^ NTA column to isolate YpeB_∆25–203_-His_6_ in addition to those proteins covalently bound via cross-links. Flow-thru (lanes 4–5) and bound (lanes 6–7) fractions were visualized via western blot using anti-YpeB antibodies [20]. The positions of protein size markers (lane 1) are indicated on the left. YpeB∆25–203 K437C monomers and dimers were detected in both the load (lane 3) and bound (lane 7) fractions of cross-linked spore samples

Attempting to the further demonstrate the possibility of a YpeB dimer, similar cross-linking experiments were performed using an allele of *ypeB*, *ypeB*_∆25–203_, that is internally truncated within the N-terminal domain, and that was previously demonstrated to produce a protein that was stably incorporated into the dormant spore [20]. Cys residues that were reactive in the previous assay were created in YpeB_∆25–203_, and the alleles were recombined into both ∆*ypeB* and WT *B. anthracis* backgrounds. Dormant spores from YpeB_∆25–203_-Cys strains were then cross-linked with APB and proteins were extracted and visualized via western blot (Fig. 2). Cross-linked extracts of YpeB_∆25–203_ K437C in a ∆*ypeB* background feature both the truncated monomer (~ 30 kDa) and what appears to be a truncated YpeB dimer (~ 60 kDa). Extracts of YpeB_∆25–203_ K437C in WT background suggest the possibility of a YpeB_∆25–203_-WT YpeB heterodimer (~ 80 kDa) in addition to complexes previously visualized. We next sought to confirm that this newly visualized band contained the 6x-His-tagged YpeB. Spores encoding YpeB_∆25–203_ K437C were cross-linked, and 6x-His-tagged proteins in extracts were concentrated using a Ni^2+^ NTA affinity column. Column elutions were visualized with western blotting (Fig. 3). The higher-migrating band (~ 60 kDa) was visualized in the cross-linked samples but was not seen in the uncross-linked controls, indicating the potential for multimerization of the YpeB C-terminal domain even in the absence of most of the N-terminal domain.

### Analyzing individual YpeB domain contributions to multimerization using bacterial two-hybrid analysis

**Fig. 4:**
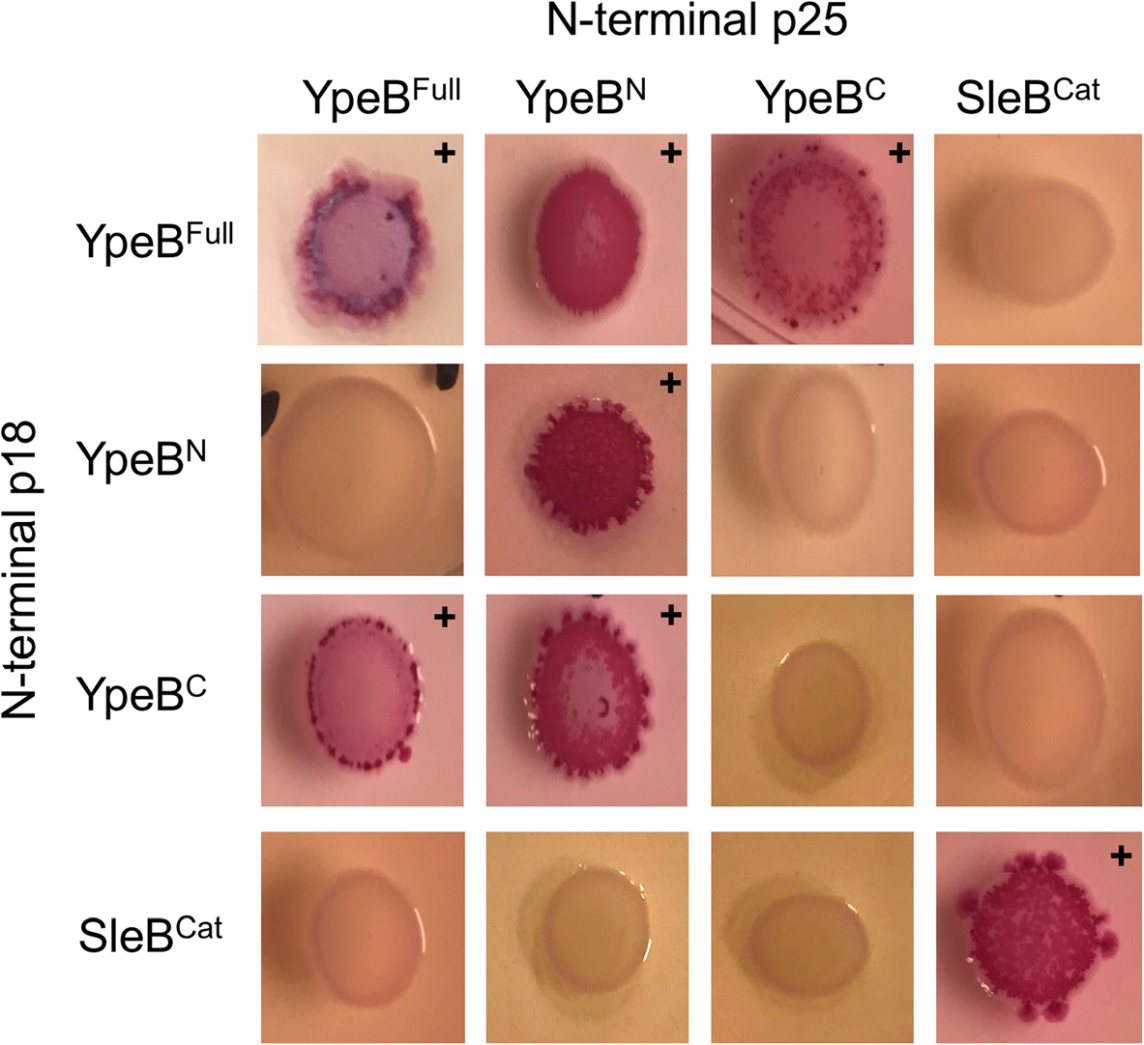
YpeB and SleB interaction detection by two-hybrid analysis. Individual domains of YpeB and SleB were inserted into both pUT18C and pKT25 creating N-terminal fusions to domains of adenylate cyclase. Plasmids were then co-transformed and screened for protein-protein interactions. Red colonies (+) indicate positive interactions. YpeB^N^ demonstrates strong self-association, while both YpeB^C^ and YpeB^N^ appear to interact with YpeB^Full^. No interactions of YpeB domains with SleB^Cat^ were detected, but SleB^Cat^ was found to interact with itself. Images are representative of three biological replicates

A bacterial two-hybrid system was implemented to better elucidate contributions of individual YpeB domains to possible multimer formation. Individual YpeB N- (YpeB^N^ 21–202) and C-terminal domains (YpeB^C^ 203–446), full-length YpeB (lacking its signal peptide) (YpeB^Full^ 21–446), and the SleB C-terminal catalytic domain (SleB^Cat^ 125–253) [28] were cloned in both pKT25 and pUT18C creating N-terminal fusions to the two domains of adenylate cyclase. Constructs were co-transformed into *E. coli*, which was plated on MacConkey agar supplemented with maltose, where positive domain interactions were visualized by red colony coloration. In agreement with cross-linking results, bacterial two-hybrid assays indicated that YpeB^Full^ self-associated. (Fig. 4). Additionally, YpeB^N^ also demonstrated self-association while YpeB^C^ did not. However, both YpeB^N^ and YpeB^C^ appear to interact with YpeB^Full^ indicating that both of these domains are involved in dimer or higher multimer structure formation. YpeB^C^ interacted with YpeB^Full^ in both orientations, while YpeB^N^ interacted with YpeB^Full^ in only one orientation of the adenylate cyclase domains. This negative result might result from this specific interaction of YpeB domains placing the fusion domains too far apart for a productive interaction. Interestingly, one orientation of the fusion domains also indicates an interaction between the YpeB^N^ and YpeB^C^ domains. Also of note, none of the YpeB constructs tested in the bacterial two-hybrid system appeared to interact with SleB^Cat^, although SleB^Cat^ did appear to associate with itself, suggesting that SleB also exists as a multimer.

## Discussion

This study further characterized interactions of YpeB within the dormant spore through in vivo chemical cross-linking and the roles of individual domains of YpeB in potential multimer formation using bacterial adenylate cyclase two-hybrid assays. Both in vivo crosslinking and two-hybrid analyses indicate that YpeB forms a dimer or higher-order multimer. The YpeB^N^ domain alone exhibits strong self-association, while the YpeB^C^ domain alone does not. However, YpeB-Cys substitutions at some positions in the C-terminal domain can be crosslinked to other YpeB molecules, suggesting close approach of C-terminal domains within the dormant spore, though without a sufficiently strong role in dimerization to be detected using the two-hybrid system. The C-terminal domain alone can interact with full-length YpeB, suggesting that either both domains are required for stable interaction with the isolated C-terminal domain, or that multimerization of the full-length protein allows further interaction with the isolated C-terminal domain.

YpeB was not demonstrated to form cross-links to SleB from selected residues within the YpeB C-terminal PepSY domains. This may indicate that the YpeB C-terminal domain, although required for stabilization of SleB [20], is not directly interacting with SleB within the dormant spore. It is also possible that these selected residues are not in correct orientation to detect an YpeB-SleB interaction. YpeB multimerization may be required for interaction or stabilization of SleB in vivo*.* Both N- and C-terminal YpeB domains have been demonstrated to be required for SleB stabilization [20, 21], however both domains may be required not because of direct interaction with SleB but rather these domains are necessary for multimer formation. Previous work demonstrated that the YpeB N-terminal can cause inhibition of SleB activity in vitro [21] and that a region beyond the first PepSY domain was required for stable incorporation of both YpeB and SleB into the spore [20]. Cross-linking data now demonstrates that residues beyond the first PepSY domain appear to be close enough to one another to form a dimer within the dormant spore. Bacterial two-hybrid analysis indicates that both the isolated YpeB N- and C-terminal domains are able to interact with full-length YpeB, thus possibly contributing to a larger multimeric structure, and N-terminal YpeB appears to strongly associate with itself, suggesting it plays a primary role in multimerization.

The structure of the YpeB C-terminal PepSY domains of *B. megaterium* has been solved [29]. Authors of the structure suggested a possible binding pocket within a channel traversing the YpeB C-terminal domain, outlined by positive charges of four lysine residues (K345, K347, K361 and K366) [29]. YpeB^S358C^, one of the residues demonstrated to be reactive in our cross-linking experiments lies directly within this potential binding-pocket. YpeB^K437C^, the other reactive residue, lies just beneath the channel but is still surface exposed. It is not immediately obvious how multiple C-terminal domains of YpeB may interact relative to this pocket.

Previous work identified conserved amino acid residues in YpeB PepSY domains required for YpeB, and subsequent SleB, stabilization in dormant spores [20]. In context of the structure of the YpeB PepSY domains, these amino acids were predicted to contribute to stabilization of the structure through intra-molecular interactions [29]. YpeB structure could be a major factor in maintaining stability of any larger multimer in which it could be involved and as such even minor disruption of its structure may ultimately lead to degradation during spore formation [20].

Although YpeB cross-linked to SleB was not detected, the list of residues tested was by no means exhaustive and it is entirely possible that the two proteins do interact, especially via the YpeB N-terminal domain [21]. YpeB may also interact with other proteins such as HtrC, which has been previously demonstrated to specifically cleave YpeB during spore germination [30]. Interaction between these, and likely other, proteins on the surface of the dormant spore membrane may serve to stabilize the proteins during long-term dormancy, and to play a key role during spore germination.

## Conclusions

The YpeB protein likely exists as a dimer or higher-order multimer in the dormant spore. Both the N- and C-terminal YpeB domains contribute to multimerization. SleB likely also exists as an oligomer, and SleB and YpeB may be found together within a protein complex. Disassembly of this complex during spore germination likely allows SleB to become active in spore cortex degradation. Further study of this protein complex may contribute to the development of methods to inhibit or stimulate germination, allowing more effective spore decontamination or more effective use of spores as applied microbial agents.

## Methods

### Strain construction

Site-directed mutagenesis by overlap extension PCR [31] was performed to create cysteine point mutants within *ypeB*. PCR products were then cloned into the *ypeB* complementation plasmid (pDPV424 [20]) via restriction-free cloning [32]. Plasmids were sequenced to verify cysteine codon substitutions and introduced into *B. anthracis* through conjugation as described previously [20, 27]. Strains with plasmid integrations (Table 1) were selected by shifting the temperature to 42 °C and verified via PCR as described previously [20].

Construction of strains for two hybrid assays was performed as follows. Desired *ypeB* and *sleB* domains were PCR amplified using primers with flanking restriction sites. PCR products were then restriction enzyme-digested along with selected vectors pUT18C and pKT25 (Euromedex). Ligations were carried out to insert *ypeB* or *sleB* domain sequences in frame with N-terminally fused p18 or p25 domains of adenylate cyclase. Plasmids were then co-transformed into BTH101 (Euromedex) to test potential interactions.

### Spore preparation

*B. anthracis* spores were prepared in liquid Modified G medium [33] with antibiotics where necessary. Spores were harvested after 3–4 days incubation at 37 °C and washed in water for several days until > 95% free of vegetative cells and cell debris. Decoated spores were prepared as described previously [20]. Briefly, spores were suspended in decoating solution (50 mM Tris-HCl pH 8, 8 M Urea, 1% SDS, 50 mM dithiothreitol) and incubated for 1 h at 37 °C. Spores were centrifuged at 8,000 x g for 2 min, and the decoating solution was removed. This procedure was repeated, followed by 5 washes with deionized water.

### Cross-linking

Ten optical density units of decoated spores were suspended in PBS pH 7.5 and APB crosslinker (Sigma) was added to a final concentration of 5 mM. Decoated spores were incubated with APB at 37 °C for 30 min in the dark. Samples were then exposed to UV light for an additional 15 min at room temperature. Following UV exposure, cross-linked spores were centrifuged at 10,000 x g for 1 min and the supernatant was removed. Cross-linked spore pellets were stored at − 80 °C until later use.

### Western blotting

Spores were lyophilized and then were broken mechanically with 100 mg 0.1 mm glass beads using Wig-L-Bug bead beaters for 20 pulses of 30 s each at 4,200 rpm. Samples were stored on ice between cycles. Proteins were extracted with 0.125 M Tris-HCl pH 6.8, 4% SDS, 10% β-mercaptoethanol, 10% glycerol, 0.004% bromophenol blue and run on SDS-polyacrylamide gel electrophoresis. YpeB and SleB were detected via western blot as described previously [20, 30]. Briefly, proteins were transferred to Amersham Hybond-P PVDF membranes (GE Healthcare). Anti-YpeB and anti-SleB antibodies were used at 1:3,000 and 1:1,000 dilutions, respectively, and horseradish peroxidase-conjugated secondary goat anti-rabbit antibodies (Bio-Rad) were used at 1:200,000 dilution. Antibody detection utilized chemiluminescence (Clarity Max Western ECL substrate; Bio-Rad).

### YpeB-His6 column chromatography

Following cross-linking of 200 OD units of decoated spores, frozen pellets were lyophilized and broken as described above. Broken spores were suspended in Urea Binding Buffer (8 M Urea, 500 mM NaCl, 50 mM Tris-HCl, 30 mM imidazole, pH 7.5) and incubated at 4 °C for 2 h. The samples were centrifuged at 6,800 x g for 10 min, and the soluble fraction was collected, filtered, and loaded onto a 1 mL Ni Sepharose HisTrap HP (GE Healthcare) column equilibrated in Urea Binding Buffer. Bound YpeB-His6 was eluted with Urea Elution Buffer (8 M Urea, 500 mM NaCl, 50 mM Tris-HCl, 1 M imidazole, pH 7.5). Fractions were stored at − 80 °C for western blot analysis.

### Bacterial adenylate cyclase two-hybrid assay

Protein interactions were screened via spotting 2 μl of co-transformed overnight culture on MacConkey agar (ampicillin 100 μg/ml, kanamycin 50 μg/ml, 1% maltose, 0.5 mM IPTG). Spotted plates were incubated for 48 h at 30 °C. Positive interactions were visualized by acidification of the media resulting in production of red coloration.

## Additional file

Additional file 1 YpeB-cysteine mutant strain functional screens. Examination of Cys-substituted YpeB protein functionality in germination rate, proteolysis during germination, assembly into the spore, and stabilization of SleB in the spore. (PDF 358 kb)

## Data Availability

All data generated or analyzed during this study are included in this published article and its supplementary information files.

## Abbreviations

APB: P-azidophencyl bromide
DPA: Dipicolinic acid
OD: Optical density
PCR: Polymerase chain reaction
SDS: Sodium dodecyl sulfate
