# Supplementary material for: YpeB dimerization may be required to stabilize SleB for effective germination of Bacillus anthracis spores
Source: BMC Microbiol. 2019 Jul 26;19:169. doi: 10.1186/s12866-019-1544-1 (PMC6660665; doi:10.1186/s12866-019-1544-1)
Supplement: Supplementary file 1 — YpeB-cysteine mutant strain functional screens. Examination of Cys-substituted YpeB protein functionality in germination rate, proteolysis during germination, assembly into the spore, and stabilization of SleB in the spore. (PDF 358 kb) [file 12866_2019_1544_MOESM1_ESM.pdf]

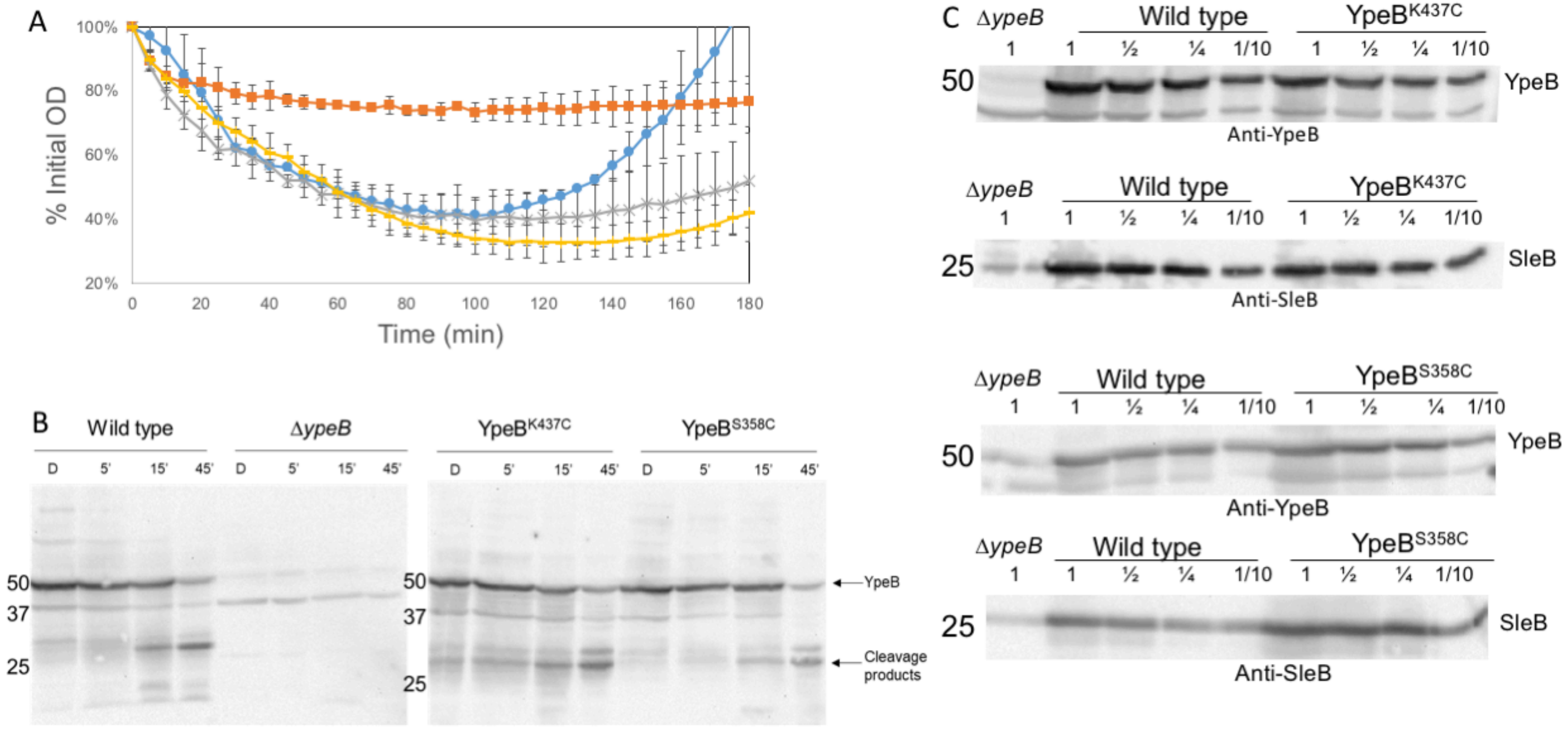

Additional File 1. YpeB-cysteine mutant strain functional screens. A. Germination rates of wild type (●), Δ*ypeB* (■), YpeB^{K437C} (+), and YpeB^{S358C} (X) spores were determined by monitoring OD_{600} change over time following addition of rich media. Spores with Cys-substituted YpeB proteins germinated similarly to the wild type but were delayed in outgrowth. **B.** Visualization of YpeB proteolytic processing during germination. Dormant spores were submitted to germination-inducing conditions, aliquots were taken at specified time points and flash frozen for later analysis via western blot. Cys-substituted YpeB proteins exhibited cleavage during germination similar to that of the wild type protein. **C.** 10 OD of dormant spores encoding YpeB-cysteine alleles and controls were lyophilized and mechanically broken. Serial dilutions of extracts were visualized via western blotting for both YpeB and SleB abundance. Cys-substituted YpeB proteins were present in spores at levels similar to that of the wild type protein and supported wild type levels of SleB accumulation.
